# Liposomal Delivery of Mycophenolic Acid With Quercetin for Improved Breast Cancer Therapy in SD Rats

**DOI:** 10.3389/fbioe.2020.00631

**Published:** 2020-06-16

**Authors:** Gopal Patel, Neeraj Singh Thakur, Varun Kushwah, Mahesh D. Patil, Shivraj Hariram Nile, Sanyog Jain, Uttam Chand Banerjee, Guoyin Kai

**Affiliations:** ^1^Laboratory of Medicinal Plant Biotechnology, College of Pharmacy, Zhejiang Chinese Medical University, Hangzhou, China; ^2^Department of Pharmaceutical Technology (Biotechnology), National Institute of Pharmaceutical Education and Research, Sahibzada Ajit Singh Nagar, India; ^3^Department of Pharmaceutics, National Institute of Pharmaceutical Education and Research, Sahibzada Ajit Singh Nagar, India; ^4^Department of Systems Biotechnology, Konkuk University, Seoul, South Korea

**Keywords:** mycophenolic acid, quercetin, anticancer, liposome, co-delivery

## Abstract

The present study explores the influence of mycophenolic acid (MPA) in combination therapy with quercetin (QC) (impeding MPA metabolic rate) delivered using the liposomal nanoparticles (LNPs). Mycophenolic acid liposome nanoparticles (MPA-LNPs) and quercetin liposome nanoparticles (QC-LNPs) were individually prepared and comprehensively characterized. The size of prepared MPA-LNPs and QC-LNPs were found to be 183 ± 13 and 157 ± 09.8, respectively. The *in vitro* studies revealed the higher cellular uptake and cytotoxicity of combined therapy (MPA-LNPs + QC-LNPs) compared to individual ones. Moreover pharmacokinetics studies in female SD-rat shown higher *T*_1__/__2_ value (1.94 fold) of combined therapy compared to MPA. Furthermore, *in vivo* anticancer activity in combination of MPA-LNPs and QC-LNPs was also significantly higher related to other treatments groups. The combination therapy of liposomes revealed the new therapeutic approach for the treatment of breast cancer.

## Introduction

Mycophenolic acid (MPA) is a secondary metabolite which is mainly produced by fungal species specialy *Penicillium brevicompactum* (Engel et al., 1982; Schneweis et al., 2000; Puel et al., 2005; Xu and Yang, 2007; Patel et al., 2016, 2017). It has diverse biological properties generally used for the treatment of rejection after organ transplant, psoriasis, and other autoimmune disorders; apart from this MPA also reported as a anticancer agent (Eugui et al., 1991; Zolezzi, 2005; Zheng et al., 2011). MPA selectively inhibit the enzyme inosine monophosphate dehydrogenase (IMPDH) in *de novo* pathway and decrease the synthesis of T cells and B cells (Eugui et al., 1991; Allison and Eugui, 2000). As per the literature, the IMPDH receptors are overexpressed in tumor cell compared to normal cells. Thus, MPA might be act as a good anticancer agent. However, many researchers also demonstrated the anticancer activity of MPA through different mechanisms (Takebe, 2006; Végso et al., 2007; Dun et al., 2013a). Even the good anticancer activity of MPA, its application rigorously stalled due to low *T*_1__/__2_ value, some undesired effects and low water solubility (Spencer et al., 1997; Shaw et al., 2001). The weak pharmacokinetic properties (lesser blood circulation time) of MPA are due to its first pass metabolism into inactive metabolites by cytochromes P450 (CYP), and UDP-glucuronosyltransferase (UGT) (Picard et al., 2004; Staatz and Tett, 2007). Very less reports are available on the delivery of MPA through nanoformulations to enhance the pharmacokinetic and pharmacodynamic properties. Liposomes can improve the pharmacokinetic of payloads. Some authors demonstrated that therapeutic liposomes can improve the pharmacokinetic of drugs thus increasing the therapeutic response of delivered drugs (Cosco et al., 2009; Paolino et al., 2010). Shirali et al. (2011) developed some soya lecithin nanoparticles for the enhancement of MPA efficacy in skin transplantation. In another study Look et al. (2013) tried to increase *in vivo* efficacy in systemic lupus erythematosus (SLE) of MPA through liposomal nanoformulation (Shirali et al., 2011). Some other researchers reported iron oxide nanoparticles of MPA for biomedical applications (Hwang et al., 2016). Ultradeformable liposomes, polymeric particles, emulsified systems, making from biocompatible materials, can be used for a successfully delivery of payloads through the skin. Some authors discussed these advantages for topical delivery of anti-inflammatory drugs (Cilurzo et al., 2015; Di Francesco et al., 2017; Critello et al., 2019). Quercetin (QC), a flavonoid abundantly found in plants having various pharmacological properties like anticancer, antiaging, hepatoprotective, and anti-inflammatory (Kashyap et al., 2016; Zhu et al., 2016). Apart from this QC also reported as an inhibitor of cytochrome P450 (CYP) enzyme which is the main enzyme of MPA metabolism (Lautraite, 2002; Tetsuka et al., 2013). The combination therapy of MPA and QC might be beneficial for cancer treatment due higher bioavailability of MPA and synergistic cell toxicity effects of both drugs. In addition, liposomal formulation of these drug can improve their pharmacokinetic properties, with higher drug accumulation in tumor and target delivery (Paolino et al., 2014; Licciardi et al., 2016). Liposome nanoparticles (LNP) are the most regularly examined nanocarrier due to their high drug loading capability, biocompatibility, self-assembling ability, and a wide range of biophysical and physicochemical properties that can be couturier to control their pharmacological features (Torchilin, 2006; Tsumoto et al., 2009; Jain et al., 2012; Chiara et al., 2017; Grillone et al., 2017; Cilurzo et al., 2019; Tan et al., 2019). On the basis of literature we try to fill out this low MPA bioavailability and efficacy gap through combination therapy of MPA and QC liposomes. Here we illustrated the preparation, extensive characterization of MPA encapsulated liposome nanoparticles (MPA-LNP) and quercetin encapsulated liposome nanoparticles (QC-LNP). The effects of individual (MPA-LNP, QC-LNP) and combined formulations (MPA-LNP + QC-LNP) with respective to control (Free MPA, free QC) were studied for cytotoxic effect in MCF-7 cell lines and female SD-rat.

## Materials and Methods

### Preparation and Characterization of Nanoparticles

MPA and QC loaded liposomes were prepared by thin-film hydration method with small alteration as per laboratory circumstances (Szoka and Papahadjopoulos, 1980). Concisely, cholesterol (1.49 mM), MPA (390 μM) and soya lecithin (1.90 mM) were dissolved in 20 ml mixture of chloroform and methanol (9:1 v/v) in a round bottom flask. Afterward, organic solvents were evaporated under reduced pressure and thin-film was obtained. Thin-film was further dried in a vacuum oven for 4 h to completely remove the traces of organic solvents. It was then hydrated at 40°C for 2 h at 100 rpm. The mixture was then probe sonicated (Mi Sonix, United States) in an ice bath to get smaller sized liposomes (Tsumoto et al., 2009; Jain et al., 2012). Similar method was used the preparation of QC liposomes, where 414 μM of QC was used.

### Physicochemical Characterization of Prepared LNPs

The prepared LNPs were characterized by dynamic light scattering (DLS) in order to determine the size and polydispersity of the liposomes using Zetasizer (Nano ZS, Malvern, United Kingdom). To confirm the morphology of the prepared LNPs, the liposomes were subjected to analyze under the transmission electron microscope (FEI Tecnai^TM^, United States) after staining the sample using phosphotungstic acid solution (2%). Further, completely dried samples of MPA, QC, and LNPs were subjected to furrier transform infrared spectroscopy (FTIR, Perkin Elmer, United States) analysis for functional group characterization. Moreover, the powdered samples (MPA, QC, liposomes etc.) were analyzed using X-ray diffractometer (D8 advanced, Bruker, United States) at a scanning rate of 10°/min over the 0–60° diffraction angle (2θ) range at 25°C in order to determine their crystallinity (Garg et al., 2015, 2016; Grillone et al., 2017; Das et al., 2019).

### Determination of Encapsulation Efficiency and *in vitro* Drug Release

The liposome solutions (1 mL) containing MPA and QC were centrifuged at 50000 rpm (4°C) for 1 h using an ultracentrifuge (Backman Coulter, United States). The supernatant from each sample was discarded and the pellet was dissolved in methanol (1 mL). The concentrations of MPA and QC in the methanolic solutions were determined using quantitative high performance liquid chromatography (HPLC) analysis (Waters, United States) (Patel et al., 2018; Thakur et al., 2019) using a C_18_ column (Symmetry^®^ C_18_, 5 μm, 4.6 × 250 mm). To quantitate the QC, acetonitrile:10 mM ammonium acetate buffer:methanol (32:48:20) smixture was used as the mobile phase. The flow rate of the mobile phase was setted up to 1 ml/min. The injection volume of the sample was 10 μl and the detection wavelength was 370 nm. Further, to quantitate MPA, water and acetonitrile (50:50 v/v) was used as the mobile phase. The flow rate was setted to 0.5 mL/min while injection volume of the MPA sample was 10 μL. The MPA sample was detected at the 220 nm wavelength. The percentage encapsulation efficiency was calculated using following equation:

(1) $$\begin{array}{c} \mathrm{Encapsulation}\;\mathrm{efficiency}\left(\%\right)=\frac{\mathrm{Amount}\,\mathrm{of}\,\mathrm{drug}\,\mathrm{in}\,\mathrm{LNPs}}{\mathrm{Total}\,\mathrm{amount}\,\mathrm{of}\,\mathrm{drug}\,\mathrm{initially}\,\mathrm{added}}\times 100 \end{array}$$

To determine the release profile of MPA and QC from liposomes, dialysis method was used. In order to study the release rate of the drugs from the developed nano formulations, the 5 mL formulation was initially centrifuged and the pellet was dispersed into the freshly prepared PBS (5 mL). The colloidal solution of the liposomes was than dialyzed against the PBS (pH 7.4) 100 mL using dialysis membrane (cut-off 10 KDa) at 37°C, 200. The 500 μL of the sample was drawn from the sink compartment at different time intervals (0, 3, 6, 12, 24, 48, and 72 h). The same amount of PBS was added to the compartment in order to maintain the volume. The quantitative analysis of the drugs into the drawn samples was performed using HPLC (Jain A. K. et al., 2013).

### Freeze-Drying of Nanoparticles

Further, the synthesized liposomes were freeze-dried in order to enhance their storage stability using a lyophilizer (Wizard 2.0, Virtis, United States) (Garg et al., 2015). Initially, 5% concentration of mannitol, trehelose and sucrose were screened as the cryoprotective agents for better stabilization of liposomes. Among these, mannitol was found to be best cryoprotective agent. In the subsequent experiment, different concentrations (2.5–10%) of mannitol were used to find out the optimum value of mannitol for maximum stability of liposome in minimum concentration of mannitiol.

### Accelerated Storage Stability

The stability of the lyophilized liposomes at accelerated storage conditions were studied for at least 6 months following the reported protocol (Garg et al., 2015, 2016). The lyophilized samples were placed into the stability chamber maintained at 25 ± 2°C and 60 ± 5% RH. The particle size and PDI of stored samples were determined after 6 months of storage into the stability chamber.

### Cell Culture Experiments *in vitro*

#### Cells

*In vitro* cell culture experiments were carried out using human breast cancer cell lines (MCF-7) procured from ATCC, United States. The preparation of media and culture conditions were setted up according to the reported ATCC protocols. In 6-well plate (Costars, Corning Inc., NY, United States), 50000 cells/well were seeded to study cellular uptake and apoptosis. However, for cell cytotoxicity analysis by MTT assay, 10000 cells/well were seeded into 96-well culture plate (Jain A. K. et al., 2013; Durán et al., 2019).

#### Cell Cytotoxicity Accessment via MTT Assay

In a 96-well plate, 10000 MCF-7 cells were seeded following the ATCC protocol and allow to attach the cells into the wells. In separate wells, media containing varied concentrations (10, 20, 40, and 60 μg/mL each) of test samples (MPA, QC, MPA + QC, MPA-LNP, MPA-LNP, QC-LNP, and MPA-LNP + QC-LNP) were added. The tetrazolium component of MTT was reduced by mitochondria present in viable cells to an insoluble formazan, however, this reduction could not be possible in dead cells. The crystals of the reduced formazan were further dissolved into DMSO and the absorbance of the solution was recorded at 540 nm (Jain A. K. et al., 2013). The cell viability having a linear relationship with the absorbance which was calculated using following equation:

Relative cell viability = (Sample Absorbance/Control Absorbance) × 100 (2)

#### Cell Uptake Study

In order to determine the cellular uptake, the cells (50000 cells/well) were allowed to attach on a 6-well plate by incubating overnight at 37°C in 5% CO_2_ environment. The cells were washed with media and attached cells were subjected to incubate with free coumarin-6 (C-6, 1 μg/mL) and C-6 loaded LNPs (1 μg/mL eq concentration of C-6) for 2 h. The media was then discarded and cells were washed using PBS (pH 7.4) twice in order to remove unentrapped C-6. The treated cells were further fixed using glutaraldehyde (2.5% v/v), washed using PBS and then subjected to confocal laser scanning microscopy (CLSM; FV1000, Olympus, Japan) (Jain A. K. et al., 2013; Velino et al., 2019).

#### Annexin V Apoptosis Assay

The cytotoxicity of the samples were tested using annexin V binding based standard phosphatidyl serin externalization assay method in treated MCF-7 cells. The cells were treated with MPA, QC, MPA + QC, MPA-LNP, QC-LNP, and MPA-LNP + QC-LNP and incubated at 37°C and 200 rpm for 6 h. The cells were then rinsed using HBSS and stained with annexin V-Cy3.18 conjugate (AnnCy3) and 6-carboxyfluorescein diacetate (6-CFDA) according to the protocol (annexinV-Cy3 apoptosis detection kit, Sigma, United States). The stained cells were analyzed under the CLSM using the red and green channels to observe the fluorescence of AnnCy3 and 6-CFDA, respectively. The quantitative determination of live, dead and apoptotic cells were performed according to the quantitative analysis of green, red, and yellow colors, respectively (Jain A. K. et al., 2013).

### Inosine-5′-Monophosphate Dehydrogenase (IMPDH) Assay

The IMPDH assay was performed to determine the enzyme inhibition activity of various test samples according to the given protocol (IMPDH assay kit, Biomedical Research Service & Clinical Application, United States). In this reaction, NADH-coupled reduction took place and iodonitrotetrazolium (INT)-formazan was formed by the reduction of INT. This formazan showed absorbance maxima at 492 nm. The quantitative analysis of INT-formazan was performed through absorbance spectroscopy. The IMPDH inhibitory activity of the test samples was determined in MCF-7 cells by incubating them with DMSO (control), free MPA (10 μM) and MPA-LNP (10 μM, eq to MPA) for 24 h (Pissinate et al., 2015).

### *In vivo* Pharmacokinetics

#### Animals, Dosing, Collection and Quantification

Female Sprague Dawley (SD) rats (200–250 g body weight) were procured from central animal facility (CAF), NIPER, SAS Nagar after the prior approval from the Institutional Animals Ethics Committee (IAEC). Following the ethical guidelines, the rats were acclimatize at 25 ± 2°C and 50–60% relative humidity under 12–12 h day and night cycle for a week. Before starting the experiments, properly acclimatized animals were kept on fasting only allowing water ‘*ad libitum*’ for overnight. The animals then randomly devided into 7 groups (5 animals/group) and animals were received MPA (25 mg/kg), QC suspension (25 mg/kg), combination of MPA and QC (25 mg/kg MPA and 25 mg/kg QC), MPA liposomes, QC liposomes and combination of MPA and QC liposomes (25 mg/kg equivalent to MPA and QC) through i.p. administration. The blood sample (250 μL) from each animal was collected in heparinized microcentrifuge tube at different time intervals (0.5, 1, 2, 6, 12, 24, and 48 h) through retroorbital plexus. The electrolyte level and central compartment volume of the animal was compensated by administering the dextrose normal saline (1 mL) orally. In centrifuge tubes containing blood samples were centrifuged at 10,000 rpm (4°C) in order to separate the blood cells and plasma. Plasma from each centrifuge tube was separated and mixed with acetonitrile (750 μL) under vortexing for 15 min in order to precipitate plasma proteins. The mixture was further centrifuged at 22,000 rpm, 4°C for another 10 min. The supernatant was collected and evaporated under reduced pressure using a rotavapor (Buchi, Switzerland). Residues of acetonitrile extract of respective samples were dissolved in methanol (100 μL). The methanolic solutions of MPA and QC were subject to quantitative HPLC analysis. (Sweeney et al., 1972; Dun et al., 2013b; Jain A. K. et al., 2013; Patel et al., 2016; Shin et al., 2019). The concentrations of the MPA and QC was plotted against the time and various pharmacokinetic parameters such as area under the curve (AUC)^0–∞^, maximum plasma concentration (C_max_) and half life (*T*_1__/__2_) were calculated using Kinetica^TM^-software (Thermo Fisher Scientific, United States) (Chaudhary et al., 2019; Dastidar et al., 2019; Hossain et al., 2019; Thakur et al., 2019).

### *In vivo* Antitumor Efficacy and Tissue Distribution

The solution of 7, 12-dimethyl[a] benzanthracene (DMBA) was prepared by dissolving it into soyabean oil. DMBA solution (45 mg/kg) was administered to the procured and acclemetized SD rats orally once per week for 3 consecutive weeks. After 10 weeks of last administration, measurable sizes of tumors into the rats were observed. Then tumor bearing rats were randomly divided in 7 groups. In the first group, saline solution was administered intraperitonially which served as control group. Other groups (test groups) received test samples namely free MPA, free QC, MPA + QC, MPA-LNP, QC-LNP and MPA-LNP + QC-LNP (25 mg/kg of MPA and QC) at each 2 days interval. The size of the tumor was calculated up to 30 days using the following formula (l × w^2^/2), where “w” was the tumor width and “l” was the tumor length. The rats were sacrificed after 30th day of study and the tumors were extracted form the body. The tumors were washed with cold PBS and their sizes were measured. Furthermore, other vital organs (heart, lung, liver, kidneys, pancreas) were also extracted. The MPA and QC quantification in various vital organs including tumors was measured in order to determine the body distribution of the MPA and QC (Dun et al., 2013b; Jain N. K. et al., 2013; Chaudhary et al., 2019; Tian et al., 2019).

### Toxicity Study

The concentrations- of blood biochemical markers were determined to check the toxicity of the liposomes after the administration. From sacrificed animals of different groups, blood specimens were collected in heparinized microcentrifuge tubes. Blood plasma was separated by centrifugation of blood at 10000 rpm, 4°C for 10 min. The concentrations of different biochemical markers [blood urea nitrogen (BUN), creatinine, aspartate aminotransferase (AST), and alanine aminotransferase(ALT)] into the plasma samples was determined using spectrophotometric method according to the specified kit protocol (Accurex Biochemical Pvt. Ltd., India) (Gao et al., 2011; Jain A. K. et al., 2013; Thakur et al., 2019).

### Statistical Analysis

The data were stated as the mean of three separate experiments with error bars shown as a standard deviation. One-way analysis of variance was carried out using GraphPadPrizm for all data sets and *P* < 0.05 was reflected statistically significant.

## Results

### Preparation of Nanoparticles and Characterization

**FIGURE 1:**
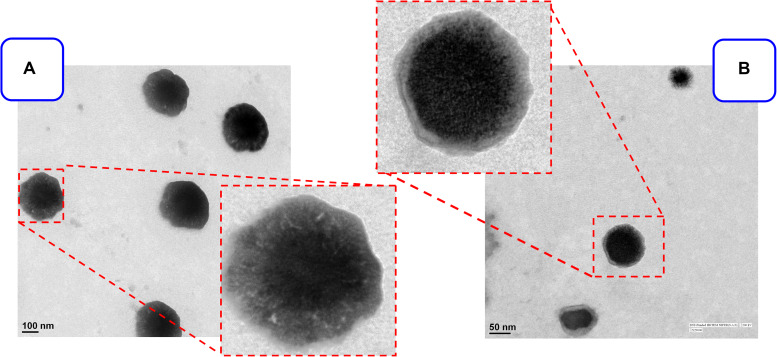
TEM images of prepared **(A)** MPA-LNP, **(B)** QC-LNP on the scale of 100 nm and 50 nm respectively.

**FIGURE 2:**
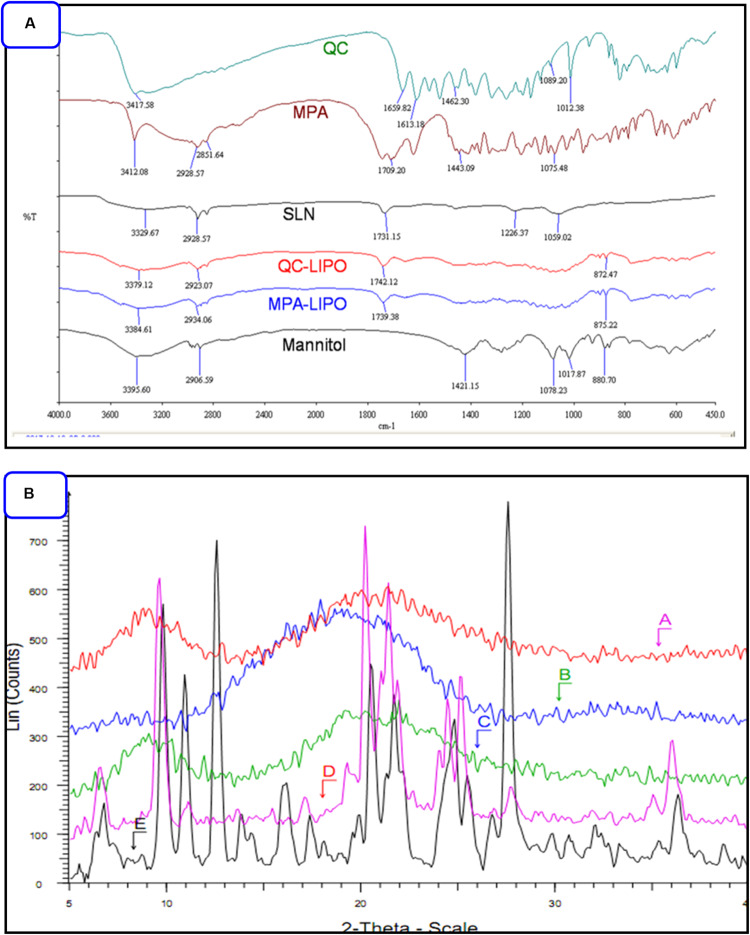
**(A)** FTIR pattern of QC, MPA, bare LNP, QC-LNP and MPA-LNP. **(B)** XRD pattern (A) MPA-LNP, (B) QC-LNP, (C) LNP without the drug, (D) QC, and (E) MPA.

The MPA and QC liposome nanoparticles were prepared and particle size and zeta potentials were found to be 183 ± 13 and 157. ± 9.8 nm, and −18.8 ± 1.3, −19.2 ± 2.6 mV, respectively of MPA-LNP and QC-LNP (Table 1 and Figure 1). The monodispersity and narrow size distribution of the prepared nanoparticles were also confirmed by polydispersity indices (PDI) which were 0.182 ± 0.020 and 0.196 ± 0.021, respectively for MPA-LNP and QC-LNP. Apart from this, TEM analysis confirmed the spherical shape and smooth-surface of MPA-LNP and QC-LNP (Figures 1A,B). FTIR analysis of various samples (QC, MPA, bare LNP, QC-LNP and MPA-LNP) revealed that there were no intermolecular interaction (Figure 2A). The graph suggested that there were no changes in the stretching and banding patterns into the spectra. Thus, it can be speculated the encapsulated MPA or QC did not interact with the functional groups of adjacent molecules and they are present in their molecular state into the liposomes. In addition, XRD analysis of all samples were also carried out and analysis revealed that there were no characteristic peaks of QC and MPA were observed in QC-LNP and MPA-LNP (Figure 2B).

**TABLE 1:** The characteristic features of prepared MPA-LNP and QC-LNP.

Parameters	MPA-LPN	QC-LPN
Average zeta size (nm)	183.40 ± 12.62	156.93 ± 09.78
PDI	0.182 ± 0.020	0.196 ± 0.021
Average zeta potential (mV)	−18.82 ± 1.34	−19.2 ± 2.61
Shape	Spherical	Spherical

### Encapsulation Efficiency and *in vitro* Drug Release of Nanoparticles

**FIGURE 3:**
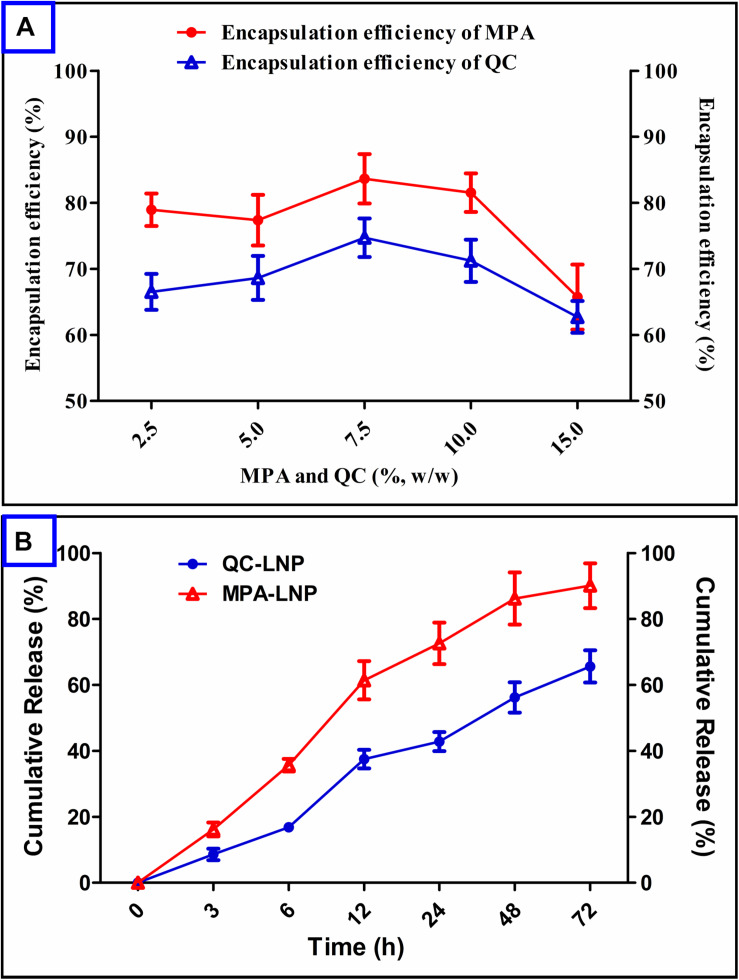
**(A)** Effect of MPA and QC concentrations on encapsulation efficiency (%) in the case of MPA-LNP and QC-LNP, **(B)** Drug release profile of MPA-LNP and QC-LNP.

Encapsulation of a drug into the nanoparticle plays very important role in further clinical applications. MPA and QC concentrations were optimized to optimum particle size and PDI and higher drug encapsulation efficiency (% EE) of nanoparticles. It has been confirmed from Figure 3A that minimum encapsulation of MPA occurred at 15 (%, w/w) and maximum at 7.5 (%, w/w) respective to soya lecithin weight. In the another hand, the maximum encapsulation efficiency of QC was found at 7.5 (%, w/w) of QC with respect to the soya lecithin weight, however, the encapsulation of MPA was found to be higher than QC. The release of MPA and QC from the prepared LNP showed in Figure 3B. The releasing rate of MPA was initially high up to 12 h in which around 60% of the MPA came out followed by a sustained release up to 48 h with the release of a total 90% MPA. Quercetin release from QC-LNP was also initially high up to 24 h in which 45% of the drug was released, however, the release rate became very slow after 24 h (Figure 3B).

### Freeze-Drying and Stability Analysis of Liposome Nanoparticles

Sucrose, mannitol and trehalose were used at the concentration of 5%, w/w of LNPs as a cyoprotectant for the long term stability and preserve the size of liposome nanoparticles as depicted in Table 2. Out these three cyoprotectant; mannitol LNP cake revealed the voluminous, easy to redisperse, fluffy and intact whereas in case of trehalose and sucrose it was not good. Insignificant difference in particle size was observed in freeze drying of LNP through mannitol although difference were significant in case of trehalose and sucrose. Afterward different concentration of mannitol (0, 2.5, 5, and 10% w/w) were also optimized for the maximum stability of LNPs. On the basis the reconstitution score and redispersibility index it was found that 5 and 10% mannitol gave the maximum accurate and stable size compared to others (Table 3). So 5% mannitol was used in the subsequent experiments in case of both MPA-LNP and QC-LNP. As per the ICH guidelines, 6 months long term stability analysis of QC-LNP and MPA-LNP were performed at 25 ± 2°C temperature and RH 60 ± 5% humidity. Conversely, in the physical appearance (shrinkage of cake) of QC-LNP and MPA-LNP insignificant difference were observed. The changes in the particle size, PDI and zeta potential of QC-LNP and MPA-LNP were also insignificant (Tables 4A,B).

**TABLE 2:** Freeze drying of MPA-LNP using various cryoprotectants at a fixed concentration (5%, w/w).

Cryoprotectants	Initial size	With freeze drying
**Mannitol**		
Size (nm)	183.40 ± 12.62	185.23 ± 13.42
Ri		1.01 ± 0.06
RS		***
**Sucrose**		
Size (nm)	183.40 ± 12.62	243.92 ± 14.56
Ri		1.33 ± 0.14
RS		*
**Trehalose**		
Size (nm)	183.40 ± 12.62	212.74 ± 19.84
Ri		1.16 ± 0.12
RS		*

Ri, redispersibility index; RS, reconstitution score, ***redispersible within 20 s with mere mixing, *reconstitution requires high shear overtaxing for 2 min. Values are presented as mean ± SD (*n* = 3).

**TABLE 3A:** Freeze drying of MPA-LNP using mannitol at different concentrations.

Cryoprotectant	Initial size	With freeze drying			
		Concentration (%, w/w)			
**Mannitol**		0	2.5	5	10
Size (nm)	183.40 ± 12.62	ND	225.58 ± 6.4	188.90 ± 13.42	187.06 ± 14.21
Ri	–	ND	1.23 ± 0.11	1.03 ± 0.06	1.12 ± 0.08
RS	–	*	**	***	***

Ri, redispersibility index; RS, reconstitution score, ***redispersible within 20 s with mere mixing, ** redispersible within 1 min, ND-not determined due to incomplete redispersion of cake. *Reconstitution requires high shear vortexing for 2 min, but the cake was not completely redispersed. Values are presented as mean ± SD (*n* = 3).

**TABLE 3B:** Freeze drying of QC-LNP using mannitol at different concentrations.

Cryoprotectant	Initial size	With freeze drying			
		Concentration (%, w/w)			
**Mannitol**		0	2.5	5	10
Size (nm)	156.93 ± 09.78	ND	183.60 ± 6.4	163.20 ± 13.42	160.06 ± 14.21
Ri	–	ND	1.17 ± 0.10	1.04 ± 0.06	1.02 ± 0.08
RS	–	*	**	***	***

Ri, redispersibility index; RS, reconstitution score, ***redispersible within 20 s with mere mixing, **redispersible within 1 min, ND-not determined due to incomplete redispersion of cake. *Reconstitution requires high shear vortexing for 2 min, but the cake was not completely redispersed. Values are presented as mean ± SD (*n* = 3).

**TABLE 4A:** Characterization of MPA-LNP after 6 months of accelerated stability studies.

Parameters	Initial	Final
Particle size (nm)	188.90 ± 13.42	188.73 ± 12.32
PDI	0.193 ± 0.02	0.191 ± 0.03
Zeta potential (mV)	−18.65 ± 1.21	−17.55 ± 1.10
Ease of reconstitution	***	***
Physical appearance	Intact fluffy cake	Intact fluffy cake

***Redispersible within 20 s with mere mixing. Values are presented as mean ± SD (*n* = 6).

**TABLE 4B:** Characterization of QC-LNP after 6 months of accelerated stability studies.

Parameters	Initial	Final
Particle size (nm)	163.20 ± 11.56	165.12 ± 14.26
PDI	0.204 ± 0.021	0.194 ± 0.018
Zeta potential (mV)	−19.4 ± 1.46	−18.5 ± 1.75
Ease of reconstitution	***	***
Physical appearance	Intact fluffy cake	Intact fluffy cake

***Redispersible within 20 s with mere mixing. Values are presented as mean ± SD (*n* = 6).

### *In vitro* Cell Culture Experiments

#### *In vitro* Cellular Uptake of LNPs and Cytotoxicity Assay

**FIGURE 4:**
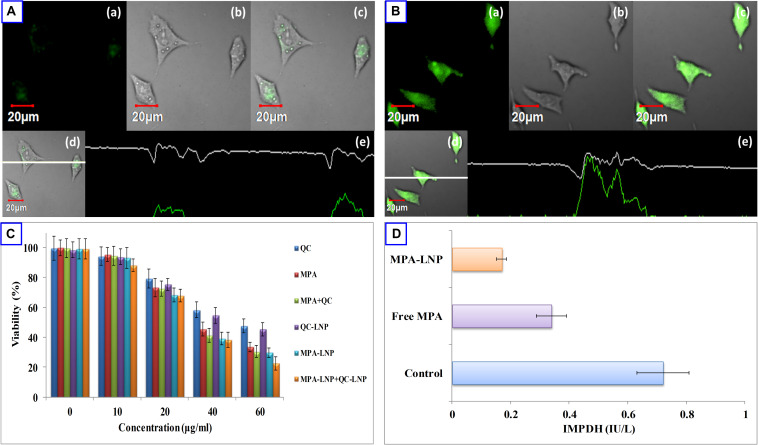
Uptake of **(A)** free C-6 and **(B)** C6-LNP by MCF-7 cells **(a)** green fluorescence of C-6. **(b)** Corresponding DIC images **(c)** overlay of **(a,b)**, and **(d,e)** vertical and horizontal line series analysis long the white line of image **(c)**. **(C)** Cell cytotoxicity of QC, MPA, combination of MPA + QC, QC-LNP, MPA-LNP and combination of MPA-LNP + QC-LNP after 24 h. Each data point represented as mean ± SD (*n* = 4). **(D)** Effect of MPA and MPA-LNP on inosine-5′-monophosphate dehydrogenase activity in MCF-7 cell lines.

The fluorescent dye C-6 was used as an indicator to label the LNPs for cell uptake analysis in MCF-7 cells. C-6 loaded LNPs (1 μg/mL, 2 h) and C-6 free LNPs were used in MCF-7 cell line for qualitative cell uptake and fluorescent intensity was measured by confocal microscopy. As depicted in Figures 4A,B after 2 h incubation of cells loaded with C-6 LNPs showed more fluorescence intensity compared to C-6 free LNPs confirming the internalization of LNPs inside the cells. *In vitro* cytotoxic potency of MPA, QC, MPA + QC, MPA-LNP, QC-LNP, and MPA-LNP + QC-LNP were investigated against human breast cancer (MCF-7) cell lines. From the Figure 4C it is clear that nanoformulation shown higher cytotoxicity compared to free drugs. The higher cytotoxicity of combined formulation (MPA-LNP + QC-LNP) revealed improved dual drug cytotoxicity compared to individual formulation (MPA-LNP and QC-LNP).

#### Annexin V Apoptosis Assay

**FIGURE 5:**
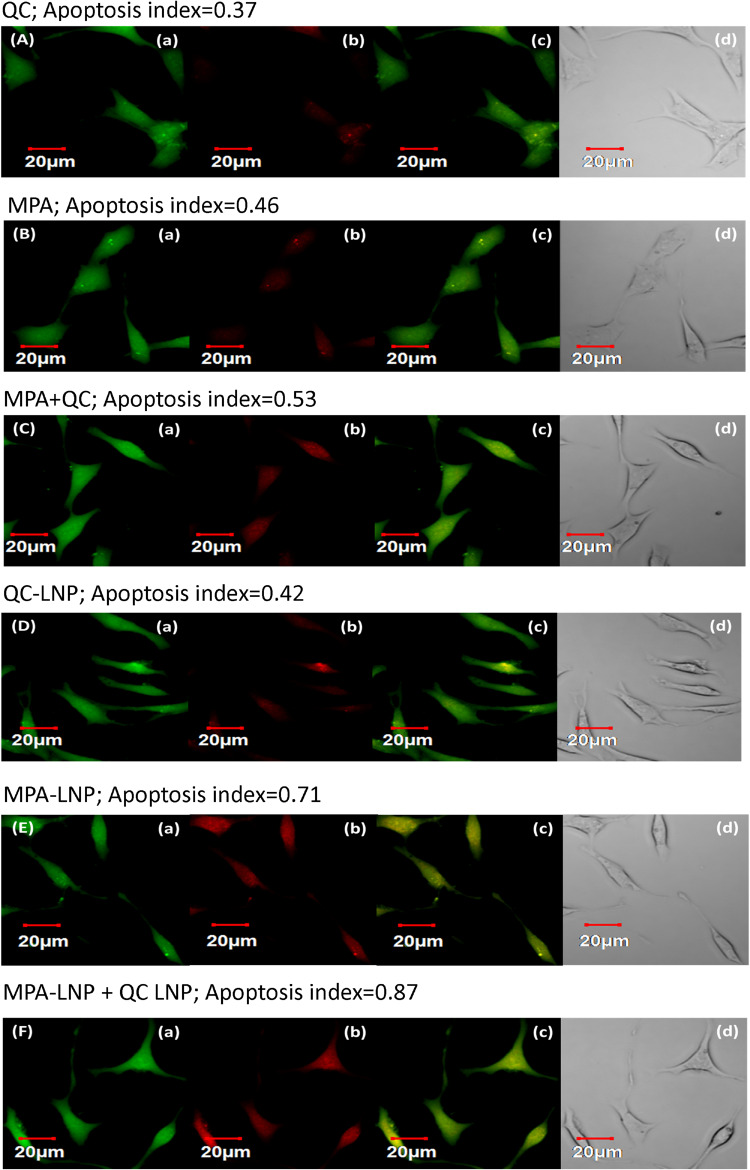
Apoptosis assay of QC **(A)**, MPA **(B)**, combination of MPA + QC **(C)**, QC-LNP **(D)**, MPA-LNP **(E)** and combination of MPA-LNP + QC-LNP **(F)** against MCF-7 cell line. **(a)** shows the green fluorescence from carboxy fluorescein (cell viability marker dye); **(b)** illustrates red fluorescence from annexinCy3.18 conjugate (cell apoptosis marker dye) **(c)** denotes the overlay image of green and red fluorescence and **(d)** depicts the differential contrast image of representative cells.

Apoptosis initiation is the principal mechanism of killing tumor cells through chemotherapic treatments so this assay was used for the further confirmation of cytotoxic effects of developed nanoformulations in MCF-7 cells. The CLSM was used for the quantitative analysis of apoptosis by calculating the apoptotic index. Figure 5 depicted the apoptotic index of different formulations (MPA, QC, MPA + QC, MPA-LNP, QC-LNP, and MPA-LNP + QC-LNP) and it compared to free MPA and QC (MPA; 0.46 and QC; 0.37) apoptosis indices significantly higher in LNPs (MPA-LNP; 0.71 and QC-LNP; 0.42). However, the maximum apoptosis index (0.87) was found in combination therapy of LNPs (MPA-LNP + QC-LNP) in comparition to free drugs combination (MPA + QC, apoptosis index 0.53). These results demonstrated the higher cytotoxicity in combination therapy of LNPs, and consistent with the consequences of MTT assay.

#### Inosine-5′-Monophosphate Dehydrogenase (IMPDH) Activity

Inosine-5′-monophosphate dehydrogenase (IMPDH) has species-specific characters catalyze inosine monophosphate (IMP) to xanthosine monophosphate (XMP) and this is the rate-limiting step of precursor synthesis of nucleotides (guanosine triphosphate). This enzyme might be a good target to cure many cancerous and autoimmune diseases due to its inhibitor sensitive effect. Therefore, quantification of IMPDH activity might be a useful tool in the disease screen and drugs designing. Some previous literature also reported that IMPDH expression is higher in cancerous cells compared to healthy, thus, it might be a good target in cancer treatment (Fellenberg et al., 2010; Majd et al., 2014). In this study, we analyzed the effects of free MPA and MPA-LNP in human breast cancer cells and measured the IMPDH activity. Figure 4D shown that the enzyme activity significantly higher in free MPA compared to MPA-LNP. Compared to control the inhibition of enzyme activities were found to be 2.11 and 4.23 folds in case of free MPA and MPA-LNP, respectively. These results demonstrated that the MPA-LNP inhibit IMPDH enzyme activity more efficiently than free MPA.

### Pharmacokinetics

**FIGURE 6:**
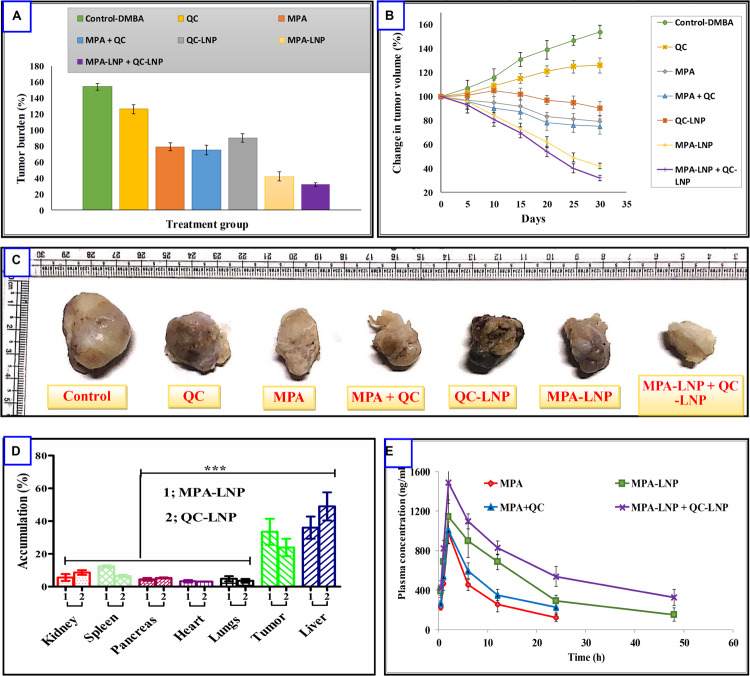
**(A)** Comparison of % change in tumor volume treated with different formulation and their combination **(B)** % tumor burden in animals treated with different formulations **(C)** representative photographs of excised tumors from different treatment groups. **(D)** Drug distribution profile of MPA and QC in the various organ of SD rat. **(E)** Plasma concentration-time profiles of MPA, MPA–LNP, MPA + QC, and MPA-LNP + QC-LNP following the i.p. administration in rats. Asterisks represents, ***P < 0.001.

Four combination of drugs (MPA, MPA + QC, MPA-LNP, and MPA-LNP + QC-LNP) administered *i.p.* in separated groups of SD rat for the analysis of *in vivo* pharmacokinetic parameters as depicted in Table 5. The AUC_(__0__–__∞__)_ and *T*_1__/__2_ values of MPA-LNP were significantly higher compared to MPA, i.e., 2.06 and 1.69 fold respectively. In second group of combination therapy, kinetic profile of MPA-LNP + QC-LNP demonstrated greater blood circulation time in the from of AUC_(__0__–__∞__)_ and T_1__/__2_, i.e., 1.34 and 1.15 fold higher correspondingly as compared to only MPA-LNP (Figure 6E). Therefore, combination therapy demonstrated 2.76 and 1.94 folds increase in AUC_(__0__–__∞__)_ and *T*_1__/__2_ values of MPA as compared to MPA alone without nanoformulation, respectively.

**TABLE 5:** Pharmacokinetic parameters of LPN upon i.p. administration in rats.

Parameters	MPA	MPA-LNP	MPA + QC	MPA-LNP + QC-LNP
AUC (ng/mL⋅h)	11533.79 ± 869.5	23768.88 ± 1395	12478.21 ± 734.2	31865.65 ± 2184.5
C_max_ (ng/mL⋅h)	979.3 ± 93	1145.29 ± 56.5	1004.38 ± 82.5	1487.39 ± 103.4
*T*_1__/__2_ (h)	13.07 ± 1.1	22.06 ± 1.6	13.79 ± 0.98	25.42 ± 2.4
MRT	18.16 ± 1.92	28.88 ± 2.4	18.84 ± 1.2	35.13 ± 2.4

*AUC, area under curve; C_max_, maximum drug concentration; T_1__/__2_, time to reach half of maximum plasma concentration; MRT, mean retention time.*

### *In vivo* Antitumor Efficacy and Tissue Distribution

Encouraged by the outstanding *in vitro* cytotoxicity results, these combination therapy were further studied in DMBA induced breast cancer animal model. As revealed in Figure 6A, combined formulation of MPA-LNP + QC-LNP showed most powerful antitumor activity and dramatically reduced the tumor burden rather than free drugs (MPA, QC) or their combination (MPA + QC) and individual formulations (MPA-LNP, QC-LNP). Figure 6B demonstrating very interesting results of different formulations effects on tumor size during 30 days of treatments which is the consistent with *in vitro* cytotoxicity and tumor burden results. As we can see in the Figure 6B, the size of tumor gradually increased in control group from 1st day (100%) to 30th day (154.29%) of treatment while in case of combination therapy its just apposite in which tumor progressively reduced from 1st day (100%) to 30th day (32.5%). After sacrificed the animals, we detached the tumor and took the picture from each groups, these tumor images clearly demonstrated that combination of two formulations shown the higher antitumor effect compared to others (Figure 6C). Furthermore, accumulation of the drugs were analyzed and compared in various organs and tumor tissue. It is clear that the maximum accumulation of MPA and QC were occurred in liver, afterward in tumor (Figure 6D).

### *In vivo* Toxicity Profile

**FIGURE 7:**
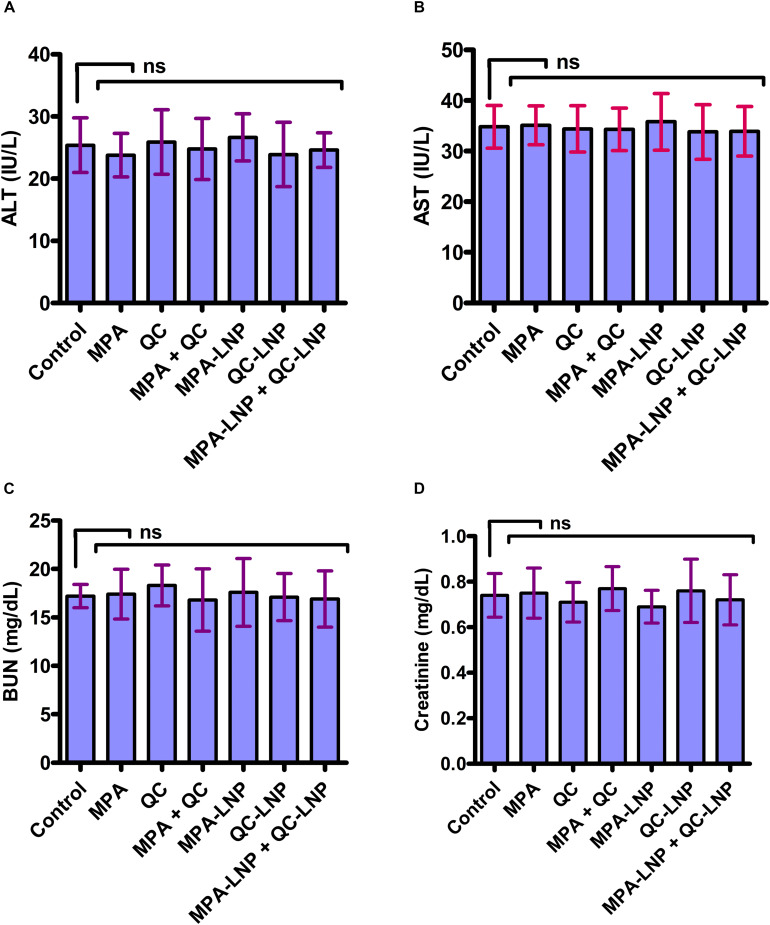
Toxicity profile of MPA, QC, MPA + QC, MPA-LNP, QC-LNP, and MPA-LNP + QC - LNP in Liver (**A**. ALT, **B**. AST) and Kidney (**C**. BUN and **D**. Creatinine).

Previous reports shown that during the conventional treatments liver toxicity is the main side effect of various drugs. For the assessment of toxicity in nanoformulations several biochemical markers have been find out (Gao et al., 2011; Jain A. K. et al., 2013; Thakur et al., 2019). *In vivo* toxicity of combine formulation was estimated using blood routine analysis, in which the concentrations of ALT, AST (hepatotoxicity biomarkers) and creatinine, BUN (nephrotoxicity) in plasma were evaluated. As shown in Figure 7, the concentration of different nephrotoxicities and hepatotoxicity biomarkers were insignificant in the rat treated with combination therapy of MPA-LNP + QC-LNP comparing to control group. These results demonstrating the minimal hepatic and renal toxicity of prepared combination therapy.

## Discussion

This study demonstrated the individual preparations of MPA-LNP and QC-LNP with their detailed characterizations. Shape and size of the prepared nanoparticles were characterized by zetasizer and transmission electron microscope (TEM). Zetasizer is works on DLS phenomena and measure the larger size of LNPs compared the TEM (Table 1). The hydrodynamic radius of the particle in the solvated state is measured in DLS technique in which solvent molecules are associated with the particle. Whereas, TEM provides the size of nanoparticles in dried form by an estimating the projected area diameter. So as far as DLS is concerned, the theory states that when a dispersed particle moves through a liquid medium, a thin electric dipole layer of the solvent adheres to its surface. This layer influences the movement of the particle into the medium. Thus the hydrodynamic diameter gives us information of the core along with any coating material and the solvent layer attached or adsorbed to the particle as it moves under the influence of Brownian motion. While estimating size by TEM, this hydration layer is not present hence, we get information only about the core and coating material (Peng et al., 2010; Su et al., 2016). Moreover, obtained results from zetasizer have a good correlation with the results of TEM (Peng et al., 2010; Bhaumik et al., 2015; Su et al., 2016; Thakur et al., 2017). FTIR studies showed that the drugs and their nanoparticles have integral properties of each component and the drugs are present as such into the LNPs (Figure 2A). XRD analysis of prepared LNP revealed that characteristic peak of free drugs completely disappeared confirming the entrapment of free drug (in their molecular form) inside the LNPs (Figure 2B) (Mu et al., 2014). Both drugs are hydrophobic in nature but encapsulation efficiency of MPA was more compared to QC at similar concentrations Figure 3A. The drug release pattern through *in vitro* hydrolysis analysis of developed nanoparticles were also carried out at pH 7.4 and releasing behavior of both drugs were biphasic, uniform, slow and controlled. In the beginning, the releasing rate of both drugs were higher due to rapid discharge of diffused and adsorbed drug from the superficial layer of LNPs (Song et al., 2009; Mei et al., 2018). Figure 3B demonstrated that the releasing rate of MPA-LNP is higher compared to QC-LNP might be due to the higher aqueous solubility of MPA at pH 7.4 than QC.

The stable network around LNPs was achieved using mannitol as a cryoprotectant and studied at different concentrations. There were no significant changes observed in the critical quality attributes of LNPs. Furthermore, after 6 months of analysis, insignificant changes were observed in the shape and size of developed LNPs. The results of the stability studies were found to be consistant with the previously reported results (Ren et al., 2018). In concentrations and time dependent manner cytotoxicity of LNPs is higher in MCF-7 cell lines related to free drugs (Figure 4C). Free drugs (MPA and QC) demonstrated the less cytotoxic effect. It may be due to less cell uptake via saturable hNTs (for MPA) and OATP1B3 (for QC) transporter and higher P-gp facilitated efflux avoiding endocytosis (Mu et al., 2014). While the greater cytotoxicity of LNPs might be due to higher stability and more drug uptake by supplementary endocytosis pathway (Su et al., 2016; Duan et al., 2017; Ren et al., 2018). Figures 4A,B, demonstrated that the internalization of C-6 loaded LNPs were higher compared to C-6 free LNPs in MCF-7 cells within 2 h of incubation. The higher internalization of liposomes might be due to clathrin- and caveolae receptor mediated endocytosis. This is the most important pathway of liposome internalization into cell because of nano size and coating of different proteins when exposed to physiological solution (Jain et al., 2014; Oh and Park, 2014). Figure 5 revealed that the apoptosis index of combination therapy (MPA-LNP + QC-LNP) is higher in comparison to alone LNPs, free drugs and their combination. The higher apoptosis index of LNPs as compared to free drugs and their combinations might be due to more cellular uptake (through clathrin-mediated) of LNPs and higher retention in the blood due to controlled release of drug for long time as mentioned in the drug release profile (Huang et al., 2019; Muniswamy et al., 2019). Moreover, the apoptosis index of combination therapy (MPA-LNP + QC-LNP) is also greater than the individual liposome formulation. This might be due to the synergistic effect of both drugs MPA and QC. In addition, the blood circulation time of MPA also increased by QC through inhibition of MPA metabolism mediated by cytochrome P450 enzyme as discussed blow in pharmacokinetic analysis (Kontermann, 2011). In addition, IMPDH enzyme inhibition activity were also carried out in MCF-7 cells using both free MPA and MPA-LNP (Figure 4B). MPA-LNPs demonstrated the higher efficacy as compared to free MPA which may be due to more internalization of liposomes inside the cells (Duan et al., 2017; Zhang et al., 2017). Pharmacokinetic analysis of MPA, MPA + QC, MPA-LNP and combination of MPA-LNP + QC-LNP were carried out in animal model which revealed that the AUC_(__0__–__∞__)_ and *T*_1__/__2_ values were found to be highest in combination of MPA-LNP + QC-LNP and lowest in case of free MPA. Higher AUC_(__0__–__∞__)_ and *T*_1__/__2_ might be due sustained release of drugs from liposomes and long blood circulation time due MPA breakdown inhibition through cytochrome P450 enzyme by QC. Previous studies reported that MPA metabolized in inactive MPAG and AcMPAG metabolites by Cytochrome P450. Thus, QC (a P450 inhibitor) might be responsible for the inhibition of metabolism of MPA and decreases the vulnerability of MPA toward the filtration or renal clearance (Kontermann, 2011). In animal model, the size of breast cancer tumor was significantly decreased in case of LNPs as compared to free MPA, free QC and free MPA + free QC. The sustained pharmacokinetic patterns of drugs, greater bioavailability, enhanced permeation and retention (EPR) effect and higher tumor accumulation of LNPs due to more uptake by clathrin and caveolae-mediated uptake might be responsible for the improved pharmacokinetic properties (Desai, 2007; Simons, 2012; Mu et al., 2014; Duan et al., 2017; Zhang et al., 2017; Mei et al., 2018; Kirar et al., 2019; Mukherjee et al., 2019). Thus, improved selective tumor cellular uptake along with EPR effect will confirm the circulation of drug-loaded LNPs in the tumor locality, which, in turn, is likely to improve the therapeutic efficacy of the loaded drugs with reduced drug-induced harmfulness (Song et al., 2009; Su et al., 2016). In addition, combination therapy of MPA-LNP + QC-LNP further decreased the tumor burden during the treatment due to synergistic effect of QC and metabolic inhibition of MPA which increased the circulation time of MPA (Lautraite, 2002; Pimple et al., 2012; Kashyap et al., 2016). These results clearly shown that combination formulation of MPA-LNP and QC-LNP provoked effective therapeutic efficacy and decrease the side effects, might be a good alternative therapy for breast cancer treatment.

In this work we have successfully developed the combination therapy of co-formulation (MPA-LNP + QC-LNP) for the treatment of breast cancer. For the treatment of breast cancer very low concentration of MPA is required due to synergistic effects of QC in this new drug combination. Meanwhile QC further improved the therapeutic efficacy by increasing the bioavailability of MPA through inhibiting Cytochrome P450 enzyme. *In vitro* and *in vivo* experiments completely demonstrate the higher anticancer effects in combination therapy (MPA-LNP + QC-LNP) compared to individual formulation or free drugs. Furthermore, formulation of MPA-LNP and QC-LNP are safe for i.p. administration and stable during free drug conversion. In summary, the combination therapy of two nano formulation realized the safe, accurate and efficient treatment of breast cancer.

## Data Availability Statement

All datasets generated for this study are included in the article/supplementary material.

## Ethics Statement

The animal study was reviewed and approved by Institutional Animals Ethics Committee (IAEC), National Institute of Pharmaceutical Education and Research, SAS Nagar.

## Author Contributions

GP, NT, GK, and UB contributed in “idea and overall outline of the work.” GP and NT performed all the experiments and wrote the manuscript. VK and SJ contributed in *in vitro* cell line studies. SN and MP contributed in characterization of synthesis. All authors contributed in data analysis and proofreading of the manuscript.

## Conflict of Interest

The authors declare that the research was conducted in the absence of any commercial or financial relationships that could be construed as a potential conflict of interest.
